# Supplementary figures and images for: Astrocytic ApoE underlies maturation of hippocampal neurons and cognitive recovery after traumatic brain injury in mice
Source: Commun Biol. 2021 Nov 18;4:1303. doi: 10.1038/s42003-021-02841-4 (PMC8602391; doi:10.1038/s42003-021-02841-4)

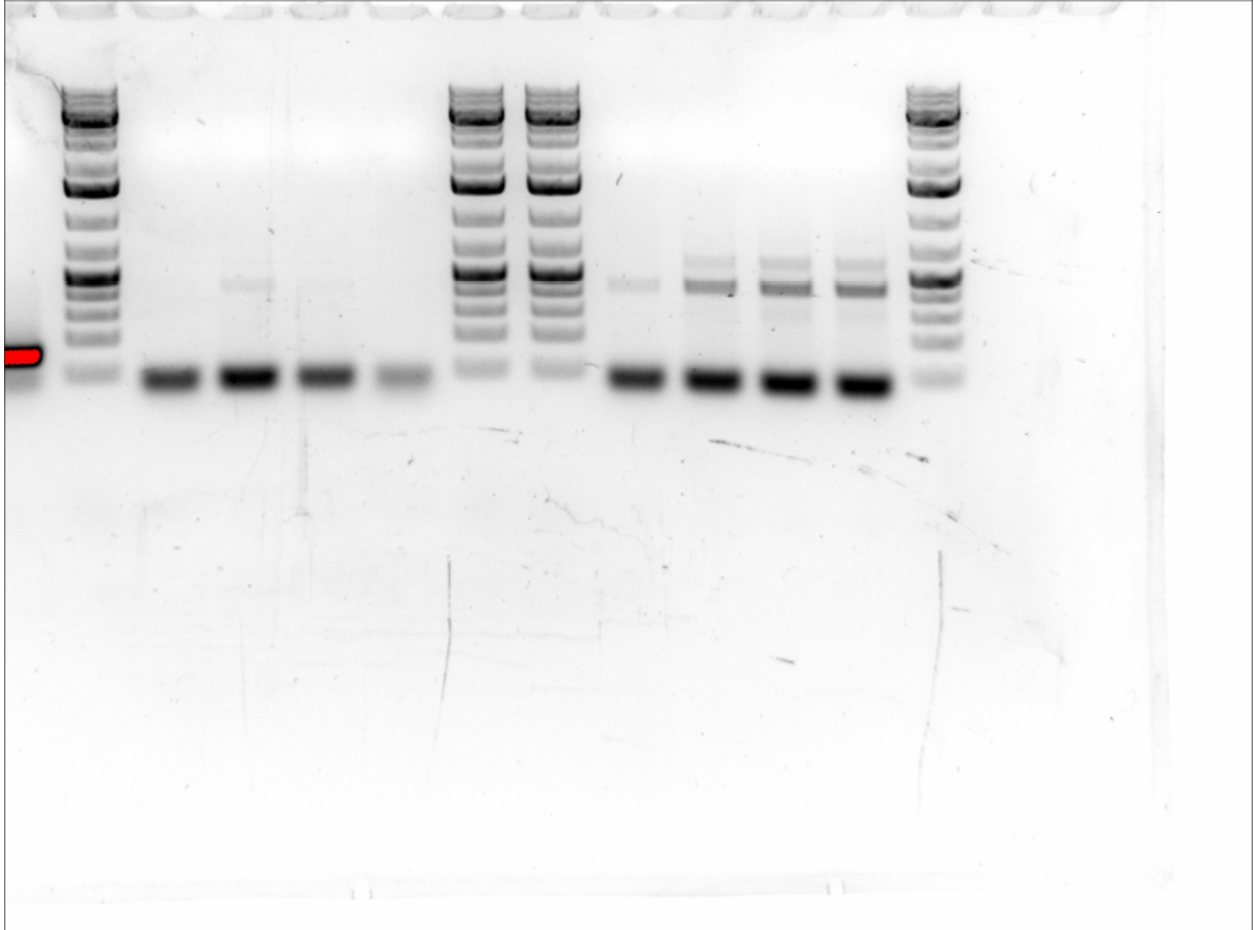

Supplement: Supplementary file 2 — Supplementary Information [file 42003_2021_2841_MOESM2_ESM.pdf]
