# Supplementary material for: Astrocytic ApoE underlies maturation of hippocampal neurons and cognitive recovery after traumatic brain injury in mice
Source: Commun Biol. 2021 Nov 18;4:1303. doi: 10.1038/s42003-021-02841-4 (PMC8602391; doi:10.1038/s42003-021-02841-4)
Supplement: Supplementary file 3 — Description of Additional Supplementary Files [file 42003_2021_2841_MOESM3_ESM.pdf]

Description of Additional Supplementary Files

File name: Supplementary Data 1.
Description: The spreadsheet has all the raw data in this study, including cell counts, normalized ApoE mRNA expression, morphological analysis, and behavioral tests.

File name: Supplementary Data 2.
Description: The statistical statement of two-way ANOVA in determining genetic and surgical effects on the complexity of dendritic trees in the pre-existing neurons in the dentate gyrus.

File name: Supplementary Data 3.
Description: The statistical statement of two-way ANOVA in determining genetic and surgical effects on the complexity of dendritic trees in the newborn neurons in the dentate gyrus.
